# Influence of Trabecular Bone Presence on Osseodensification Instrumentation: An In Vivo Study in Sheep

**DOI:** 10.3390/biomimetics9090568

**Published:** 2024-09-19

**Authors:** Zachary Stauber, Shangtao Wu, Justin E. Herbert, Amanda Willers, Edmara T. P. Bergamo, Vasudev Vivekanand Nayak, Nicholas A. Mirsky, Arthur Castellano, Sinan K. Jabori, Marcelo V. Parra, Estevam A. Bonfante, Lukasz Witek, Paulo G. Coelho

**Affiliations:** 1 University of Miami Miller School of Medicine, Miami, FL 016960, USA; 2 Department of Biochemistry and Molecular Biology, University of Miami Miller School of Medicine, Miami, FL 33136, USA; 3 Operative Dentistry Division, Department of Restorative Dentistry, Piracicaba Dental School, Piracicaba, State University of Campinas, Piracicaba 13414-903, Brazil; 4 Biomaterials Division, NYU Dentistry, New York, NY 10010, USA; 5 Mackenzie Evangelical School of Medicine Paraná, Curitiba 80730-000, Brazil; 6 Federal University of Parana, Curitiba 80060-000, Brazil; 7 Division of Plastic Surgery, DeWitt Daughtry Family Department Surgery, University of Miami Miller School of Medicine, Miami, FL 33136, USA; 8 Center of Excellence in Morphological and Surgical Studies (CEMyQ), Faculty of Medicine, Universidad de la Frontera, Temuco 01145, Chile; 9 Department of Comprehensive Adult Dentistry, Faculty of Dentistry, Universidad de la Frontera, Temuco 01145, Chile; 10 Department of Prosthodontics and Periodontology, Bauru School of Dentistry, University of Sao Paulo, Bauru 17012-901, Brazil; 11 Hansjörg Wyss Department of Plastic Surgery, NYU Grossman School of Medicine, New York, NY 10016, USA; 12 Department of Biomedical Engineering, NYU Tandon School of Engineering, 6 MetroTech, Brooklyn, NY 11201, USA

**Keywords:** osseodensification, implants, additive instrumentation, osteotomy, trabecular bone

## Abstract

Osseodensification enhances the stability of endosteal implants. However, pre-clinical studies utilizing osseodensification instrumentation do not account for the limited presence of trabeculae seen clinically. This study aimed to evaluate the effect of osseodensification instrumentation on osteotomy healing in scenarios with and without the presence of trabecular bone. A ~10 cm incision was made over the hip of twelve sheep. Trabecular bone was surgically removed from twelve sites (one site/animal; negative control (Neg. Ctrl)) and left intact at twelve sites (one site/animal; experimental group (Exp.)). All osteotomies were created using the osseodensification drilling protocol. Each osteotomy received an endosteal implant and was evaluated after 3 or 12 weeks of healing (n = 6 animals/time). Histology revealed increased woven and lamellar bone surrounding the implants in the Exp. group relative to the Neg. Ctrl group. The Exp. group demonstrated the presence of bone fragments, which acted as nucleating sites, thereby enhancing the bone formation and remodeling processes. Bone-to-implant contact (%BIC) and bone area fractional occupancy (%BAFO) were significantly higher in the Exp. group relative to the Neg. Ctrl group both at 3 weeks (*p* = 0.009 and *p* = 0.043) and 12 weeks (*p* = 0.010 and *p* = 0.008). Osseodensification instrumentation in the presence of trabecular bone significantly improved osseointegration. However, no negative influences such as necrosis, inflammation, microfractures, or dehiscence were observed in the absence/limited presence of trabeculae.

## 1. Introduction

Tooth loss occurring from iatrogenic, traumatic, or therapeutic causes often leads to edentulism [1], therefore necessitating the utilization of implants and their subsequent anchorage for long-term rehabilitation [2,3]. While effective, one of the most common complications is implant screw loosening, estimated to affect up to ~12% of patients [4,5,6], which is due to a variety of clinical scenarios (i.e., technical errors during positioning, screw length, inappropriate mechanical transmission of stress on the device, and/or poor bone quality) [7,8,9]. Furthermore, the frequency of early implant failure increases with additional factors such as smoking, the absence of post-operative antibiotic therapy, bone augmentation, and implant dimensions. Procedures to address implant failure can be financially prohibitive endeavors and potentially difficult technical procedures due to the scarred, distorted anatomy and, in certain circumstances, the need to use specialized instruments [10,11].

Several different approaches, such as the modification of screw length, diameter, or insertion trajectory; fenestration design; and osteotomy preparation techniques, have been employed to resolve implant failure [12,13]. For successful implant placement, adequate bone compression around the screw upon immediate insertion and long-term screw fixation (primary and secondary stability, respectively) are paramount [14]. Primary stability is essential to long-term success as it prevents the micromotion of the implant during the early stages of the healing process. The degree of primary stability can be influenced by implant design, osteotomy size, bone density, and/or patient comorbidities [15]. Increased levels of primary stability at the time of implant insertion have been shown to elicit rapid secondary stability [16]. This necessitates maximizing primary stability at the time of endosteal implant insertion to thereby increase the probabilities of long-term implant fixation. 

A potential option for maximizing primary stability is increasing bone-to-implant contact [17]. Instrumentation (e.g., drilling technique) is a primary aspect to be considered when high primary stability is desired. Several instrumentation modalities have been suggested to increase primary stability, particularly in low-density bone [18,19,20]. However, the introduction and implementation of additive instrumentation techniques (e.g., osseodensification) to improve osseointegration has repeatedly demonstrated favorable results in attempts to minimize implant loosening by maximizing primary stability and osteointegration [21,22]. To elaborate, osseodensification is a form of ‘additive instrumentation’ whereby the burr compacts the bone fragments into the osteotomy wall [23] and the preserved bone chips (e.g., autografts) act as nucleating surfaces at the bone-to-implant interface, facilitating bone formation and osseointegration [24,25].

The efficacy and viability of osseodensification instrumentation relative to conventional subtractive techniques within uncompromised (native) trabecular bone have been established [22]. Osseodensification sub-instrumentation, the use of additive instrumentation in the context of different macro-geometries, and the surface parameters of implants have also been explored [26]. However, a systematic review in 2020 by Padhye et al. indicated that while osseodensification may be particularly useful in areas with relatively high amounts of cancellous bone, its use in areas with limited trabecular bone (primarily corticated bone) warranted evaluation due to a dearth of published data [24]. Yet, pre-clinical and clinical studies in the literature focusing on the use of osseodensification instrumentation have largely not accounted for limited trabecular bone volume at the osteotomy site [27]. 

In addition, a study by Koutouzis et al. suggested that osseodensification in sites with a limited volume of trabecular bone may produce a higher risk of bone overstraining and microfractures, although this has yet to be established through pre-clinical or clinical evaluation [28]. Building on these findings and assumptions, the objective of the current study was to evaluate the bone regeneration outcomes in the immediate vicinity of endosteal implants inserted into osteotomy sites prepared using osseodensification drilling in the limited presence of underlying trabecular bone.

## 2. Methods

### 2.1. Pre-Clinical Experiments

**Figure 1:**
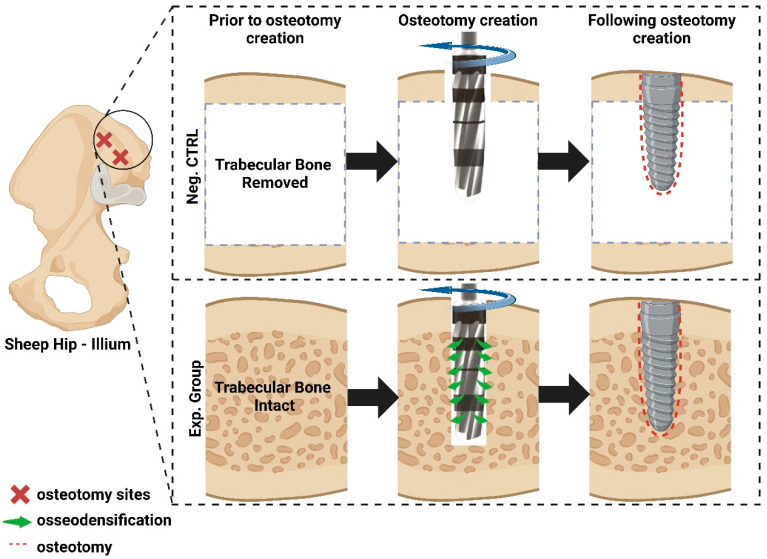
Schematic of the 2 groups (Neg. Ctrl and Exp.) used in this study. Image generated on Biorender.com.

Upon approval from École Nationale Vétérinaire d’Alfort (Maisons-Alfort, Ile-de-France, France) Institutional Animal Care and Use Committee (IACUC), 12 adult sheep [n = 6 per time in vivo (3 and 12 weeks)] were obtained and allowed to acclimate for approximately one week. All surgical procedures were performed under an aseptic environment and general anesthesia. Each animal was injected with sodium pentathol (15–20 mg/kg) in a Normasol solution in the jugular vein. Anesthesia was maintained with isoflurane (1.5–3%) in O_2_/N_2_O (50/50). Concurrently, ECG, SpO_2_, and final tidal CO_2_ were used to track vital signs. The ilium was selected as the site for osteotomy and implant placement. At the time of surgery, the site was shaved and prepared with an iodine solution, followed by an incision of ~10cm in the anteroposterior direction over the iliac crest. Subsequently, iliac bone was exposed and 2 osteotomies were prepared as follows: (1) with trabecular bone (Exp.) and (2) limited bone (Neg. Ctrl) in the trabecular space; i.e., trabecular bone was surgically removed/deburred from the iliac crest between the cortical plates at the site of surgery, leaving the cortical bone thickness intact prior to osseodensification drilling at a total of twenty-four sites (Figure 1).

**Figure 2:**
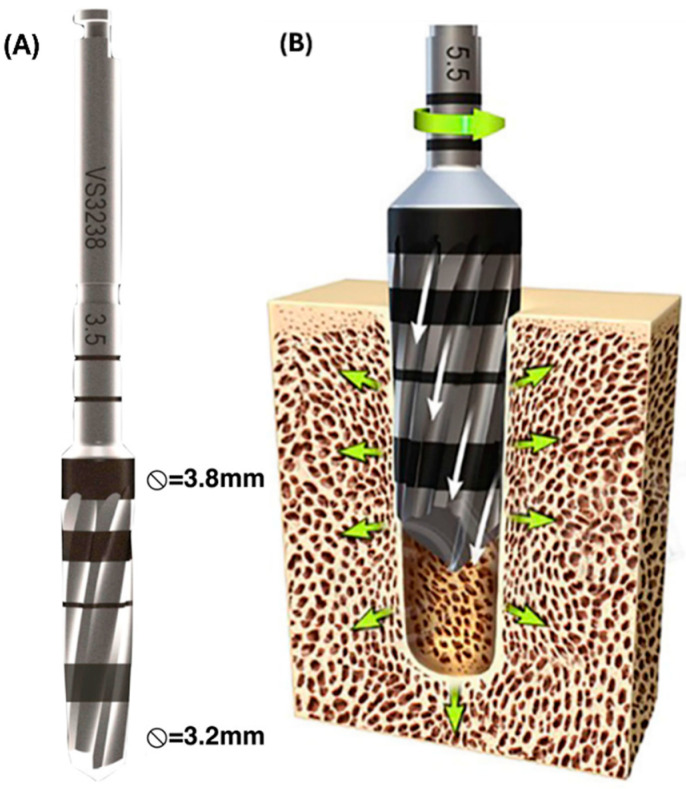
(**A**) Computer-Aided Design images of Densah^®^ multifluted tapered burs (Versah LLC, MI, USA). Reproduced with permission from [26], copyright 2018 Orthopaedic Research Society. Published by Wiley Periodicals, Inc. (**B**) Schematic picture of osseodensification drilling method (Image courtesy of Versah LLC, MI, USA). Reproduced with permission from [29], copyright 2020 Orthopaedic Research Society. Published by Wiley Periodicals LLC.

Osseodensification drilling (Figure 2) was performed in a counterclockwise fashion using the Densah^®^ multifluted tapered bur (Versah LLC., Jackson, MI, USA) 2.0 mm pilot drill, followed by the 2.8 mm and 3.8 mm burs at 1100 rpm under continuous saline irrigation, after which each osteotomy received a Ti-6Al-4V screw root form endosteal implant (Emfils, Itu, Brazil)—4 mm in diameter, 10 mm in length, and with a machined (regular) surface with no additional surface treatments. All implants were torqued into the osteotomy as per the manufacturer’s specifications (85 N.cm ± 10%).

Subjects were randomly allocated to one of two healing times, 3 or 12 weeks (n = 6/time). Surgical sites were sutured using Vicryl 2-0 for muscle and nylon 2-0 for skin. Cefazolin (500 mg) was administered pre-operatively and post-operatively via intravenous injections to reduce the appearance of post-operative complications. Animals were provided food and water ad libitum. Three and twelve weeks after the first surgical intervention, animals were euthanized according to the approved protocol and samples were harvested en bloc.

### 2.2. Histomorphometric Analysis

The bone–implant blocks were gradually dehydrated in a series of alcohol solutions ranging from 70% to 100% ethanol. Following dehydration, the samples were immersed in a clearing solution (methyl salicylate) and subsequently embedded in a methacrylate-based resin. The embedded samples were cut into slices (~300 μm) with a precision diamond wafering saw (Isomet^®^ Low Speed, Buehler Ltd., Lake Bluff, IL, USA). The slices were generated transversally (relative to the long axis of the implant), such that the implant cross-section was visible.

**Figure 3:**
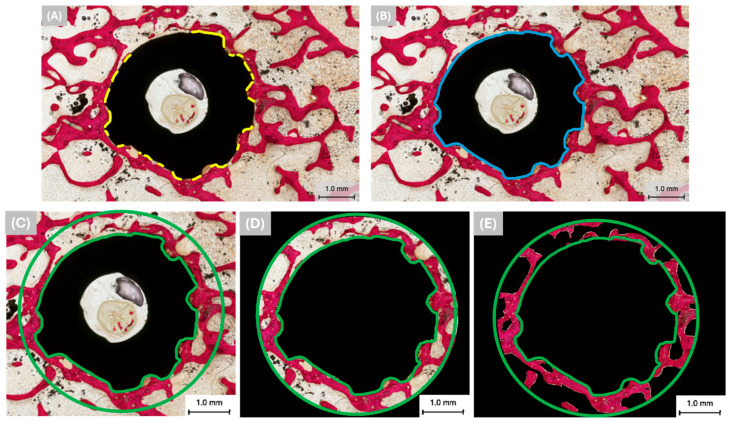
Representative histomicrographs showing (**A**) the perimeter of the implant surface covered with new bone (yellow lines), (**B**) the total transverse perimeter of the implant (blue lines), (**C**) the region of interest (ROI) in green highlights, (**D**) the total area encompassed within the ROI, and (**E**) the bone area within the ROI. Bone is colored red due to SVG staining.

Sections were glued to acrylic slides with a cyanoacrylate-based adhesive (Loctite 408, Henkel AG, Dusseldorf, Germany), and a 24-h setting time was allowed prior to grinding and polishing. The sections were then reduced to a final thickness of ~80 μm by means of a series of silicon carbide (SiC) abrasive papers (400, 600, 800, and 1200 grit; Buehler Ltd., Lake Bluff, IL, USA) using a grinding/polishing machine (Metaserv 3000, Buehler Ltd., Lake Bluff, IL, USA) under copious water irrigation. Subsequently, the samples were stained with Stevenel’s Blue and Van Gieson’s Picro Fuchsin (SVG) and digitally scanned via an automated slide scanning system (Aperio CS2, Vista, CA, USA) and specialized computer software (Aperio ImageScope 12.4.6, Vista, CA, USA). Stevenel’s Blue stained cells and extracellular structures in a subtle gradation of blue tones. The counterstain, Van Gieson’s Picro Fuchsin, stained collagen fibers green or green-blue; bone in red, orange, or purple; and muscle fibers in blue to blue-green. Bone-to-implant contact (%BIC) in the cancellous layers was quantified (one slide from each implant/osteotomy was chosen) using image analysis software (ImageJ 1.54h, NIH, Bethesda, MD, USA) as per the following equation (and illustrated in Figure 3A,B):

$$\%BIC=\frac{The\;perimeter\;of\;the\;implant\;surface\;covered\;with\;new\;bone\times 100}{The\;total\;perimeter\;of\;the\;implant}$$

On the other hand, bone area fractional occupancy (%BAFO) was used to quantify the osteogenic parameters in the region of interest (ROI). The ROI is defined as the area between the green highlights in Figure 3C. To elaborate, the green circle is up to 1 mm away from the implant surface. %BAFO was quantified (one slide from each implant/osteotomy was chosen) in the cancellous layers using image analysis software (ImageJ 1.54h, NIH, Bethesda, MD, USA) according to the following equation (and illustrated in Figure 3C–E):

$$\%BAFO=\frac{Bone\;area\;within\;the\;ROI\times 100}{Total\;area\;encompassed\;within\;the\;ROI}$$

**Figure 4:**
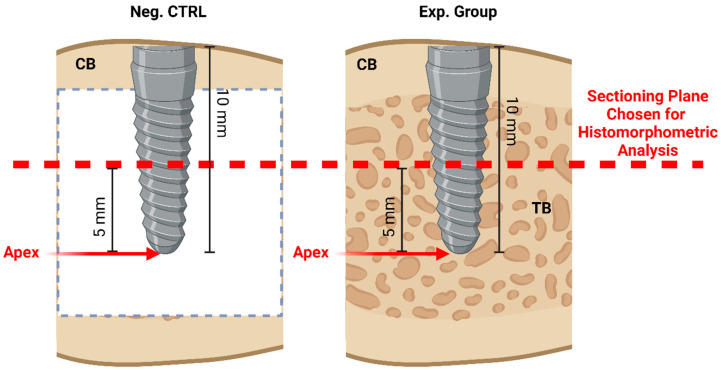
Schematic illustration of the histological sectioning plane (in dashed red lines) chosen for histomorphometric analyses. CB = Cortical Bone; TB = Trabecular Bone. Image generated on Biorender.com.

The choice of section plane has been suggested to have an influence on the histomorphometric findings [30]. However, it has been established that the same cutting/sectioning plane can be taken into account to quantify the bone growth around the implant to standardize the results [30]. As such, to ensure that %BIC and %BAFO values are comparable between implants and/or test subjects, a standardized selection of the sectioning plane was performed. Once the sectioning plane (transverse to the implant longitudinal axis) was defined to be located 5 mm from the apex of the implant (illustrated in Figure 4), it was kept consistent among all the slides analyzed to minimize bias.

### 2.3. Statistical Analysis

Statistical analysis was performed using IBM SPSS (v29, IBM Corp., Armonk, NY, USA), with histomorphometry data presented as mean values with 95% confidence interval values (mean ± 95%CI). The assessment of the normality of the data is a prerequisite for parametric statistical tests given underlying assumptions. As such, due to the sample size, the Shapiro–Wilk test was performed to confirm data normality (*p* > 0.05), prior to the use of an appropriate statistical analysis. The values of %BIC and %BAFO were analyzed with a linear mixed model (analogous to logistic regression and optimized for nested within subject observations) and fixed factors of surgical instrumentation method (Neg. Ctrl and Exp.) and time in vivo (3 and 12 weeks).

## 3. Results

During the immediate post-operative evaluation, no surgical site revealed any sign of inflammation or infection, and there was no evidence of implant failure at the time of necropsy.

### 3.1. Qualitative Histologic Findings

**Figure 5:**
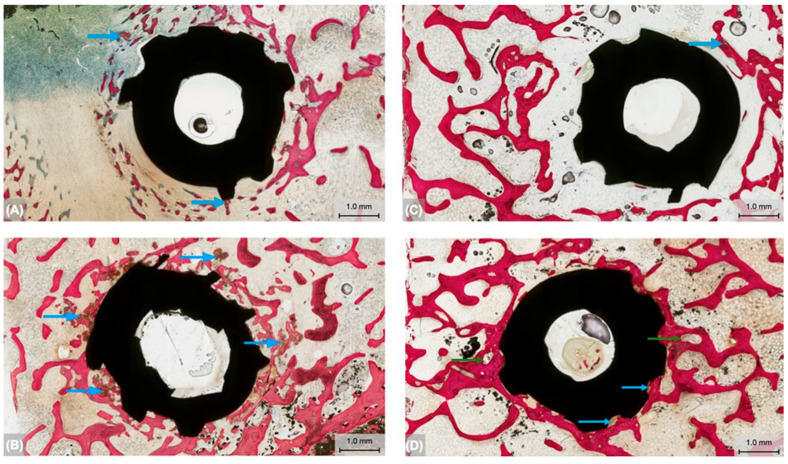
Representative histomicrographs of the Neg. Ctrl and Exp. groups at (**A**,**B**) 3 and (**C**,**D**) 12 weeks, respectively. Blue arrows represent the bone fragments compacted around the implant as a result of osseodensification instrumentation. Green arrows point toward bone remodeling sites in the immediate vicinity of the implant in the Exp. group.

Bone formation around the endosteal implants was qualitatively analyzed at the two healing time points. At 3 weeks, the Neg. Ctrl group (Figure 5A) demonstrated a limited presence of newly formed woven bone in the immediate periphery of the implant. Relative to the Neg. Ctrl group, the Exp. group (Figure 5B) presented with increased degrees of woven bone surrounding the implant, suggesting greater primary stability at the early healing time point. Additionally, the osseodensification instrumentation in the presence of the trabecular network resulted in the compaction of a larger number of bone chips towards the osteotomy walls (blue arrows, Figure 5B) surrounding the immediate vicinity of the implant. Bone chips were attached and embedded into the osteotomy wall and the implant surface.

At the extended healing time, 12 weeks, the Neg. Ctrl group (Figure 5C) revealed increased bone formation with indications of lamellar reorganization relative to the 3-week time point. The Exp. group (Figure 5D) presented higher degree of new bone formation surrounding the implant. Furthermore, bone formation in the Exp. group was more pronounced at this advanced healing time point relative to 3 weeks, with the new bone surrounding the implant appearing lamellar and progressing toward advanced degrees of remodeling (green arrows, Figure 5D). Similar to the early healing time point, bone chips as a result of the osseodensification process in the Exp. group were found to be embedded into the osteotomy wall and the implant surface (blue arrows, Figure 5D), seemingly acting as autologous grafting particles owing to their encapsulation by the remodeling bone.

### 3.2. Histomorphometric Analysis

**Figure 6:**
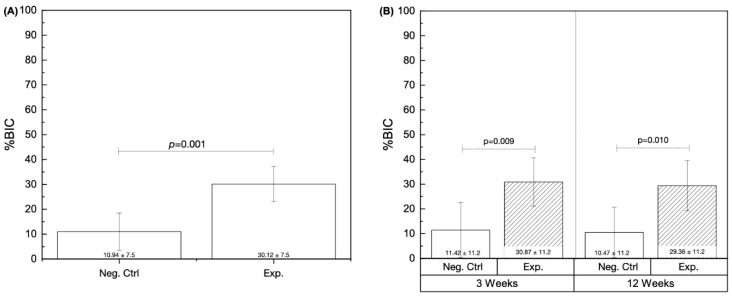
Histomorphometric data. (**A**) %BIC collapsed over time (3 and 12 weeks), and (**B**) %BIC as a function of experimental group and time. *p* < 0.05 is statistically homogeneous.

The quantitative evaluation of bone-to-implant contact (%BIC) independent of time in vivo yielded significantly higher results in the Exp. group (30.12% ± 7.5) relative to the Neg. Ctrl group (10.94% ± 7.5) (*p* = 0.001) (Figure 6A). Subsequent analyses of %BIC as a function of drilling technique and time in vivo detected statistical differences at 3 weeks (*p* = 0.009) and 12 weeks (*p* = 0.010), where the Exp. group [30.87% ± 11.2 (3 weeks) and 29.36% ± 11.2 (12 weeks)] yielded higher values relative to the Neg. Ctrl group [11.42% ± 11.2 (3 weeks) and 10.47% ± 11.2 (12 weeks)] (Figure 6B).

**Figure 7:**
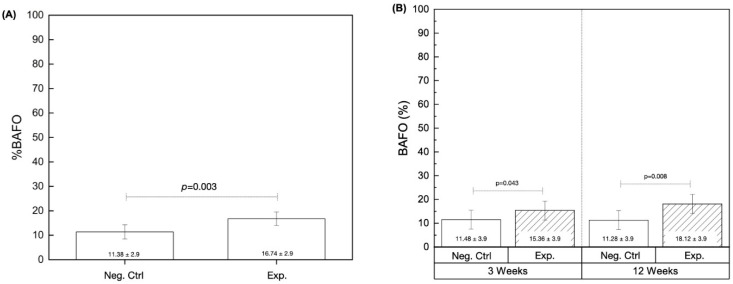
Histomorphometric data. (**A**) %BAFO collapsed over time (3 and 12 weeks), and (**B**) %BAFO as a function of experimental group and time. *p* < 0.05 is statistically homogeneous.

The evaluation of bone area fractional occupancy (%BAFO) independent of healing time was significantly higher in the Exp. group (16.74% ± 2.9) in comparison to the Neg. Ctrl group (11.38% ± 2.9) (*p* = 0.003) (Figure 7A). Additionally, %BAFO results as a function of instrumentation technique (Neg. Ctrl vs. Exp.) and time in vivo (3 vs. 12 weeks) revealed statistical differences. For instance, %BAFO was higher in the Exp. group (15.36% ± 3.9) relative to the Neg. Ctrl group (11.48% ± 3.9) at 3 weeks (*p* = 0.043). A similar trend was observed at the advanced healing time point, where the Exp. group yielded higher %BAFO (18.12% ± 3.9) in comparison to the Neg. Ctrl group (11.28% ± 3.9) (*p* = 0.008) (Figure 7B).

## 4. Discussion

Conventional, subtractive drilling instrumentation pertaining to implant fixation has been utilized and is extensively available in the literature. Nevertheless, its limitation (i.e., excavating bone at the site of the osteotomy) has been shown to negatively impact bone regeneration, with remodeling ultimately resulting in the loss of viable bone fragments at the bone–implant interface which have the capacity to bridge the gap between the osteotomy walls and the implant surface, thus necessitating the use of additive instrumentation techniques such as osseodensification. Studies pertaining to osseodensification instrumentation have highlighted its efficacious bone regenerative capabilities [22,26]. However, the aforementioned studies that outline the use of osseodensification in pre-clinical settings have not elucidated its effect on bone regeneration around endosteal implants placed in areas with limited trabecular volume.

In this context, the current study examined the effect of osseodensification drilling with and without trabecular bone in a large translational model. Utilizing the sheep model for this study and selecting the ilium due to its low-density bone configuration allowed for the experimental groups to be nested within each animal owing to its size, thereby maximizing statistical power while minimizing the number of animals used for experimentation. Additionally, using low-bone-density sites such as the hip effectively simulated the low-bone-density settings seen clinically.

The qualitative result of the current study strongly indicates that osseodensification drilling did not have any negative influence (i.e., necrosis, inflammation, or dehiscence) on bone healing. The quantitative histomorphometric analyses confirmed that healing outcomes were significantly greater in the presence of trabecular bone volume at both the early (3-week) and advanced (12-week) healing time points. Pertaining to the Exp. Group, contained within the trabecular bone, between the layers of cortical bone, is a porous network of bone cells and marrow [31]. This sponge-like cellular network has been shown to result in an increased surface area of bone cells relative to cortical bone [32], which not only elicits greater bone formation but also facilitates osseodensification instrumentation.

The osseodensification burs possess a large negative rake angle and act as non-cutting edges to allow for the compaction of bone fragments, thereby increasing bone density at the site of the osteotomy. Das et al. [33] described the mechanism of the osseodensification drilling technique in which counterclockwise rotation compacts bone particles into the trabecular wall. Once the bone particles are coated to the osteotomy walls, they promote increased bone density at the bone–screw surface while also promoting primary stability and, as a result, increased osseointegration [34]. This was confirmed through the qualitative histomicrographs of the Exp. group at both time points, seen as the preservation of the bone bulk through the enhancement in bone density by the lateral compaction or displacement of autografting bone particles at the walls of the osteotomy. Osseodensification was seen to preserve the bone-chip autografts which acted as nucleating surfaces at the bone–implant interface, ultimately facilitating osseointegration, as seen previously [26]. Such a phenomenon was not observed in the Neg. Ctrl group, largely owing to the lack of trabecular bone volume, highlighting the requirement of trabecular bone at the site of the osteotomy to achieve sufficient osseodensification using Densah^®^ multifluted tapered burs.

On the other hand, pertaining to the comparison of osseodensification instrumentation to conventional subtractive (osteotomy) techniques, have been reported by Buchter et al. that the latter hampers bone remodeling and causes ultrastructural microdamage, and that the biomechanical stability may be significantly decreased shortly after implant placement [35]. This could be attributed to microcracking at the osteotomy sites due to strain that exceeds bone’s elasticity. Furthermore, in another study by Alifarag et al., histologic micrographs around the osseodensification instrumented implants demonstrated a lower incidence of microcracking due to compression relative to conventional drilling [26]. While the current study qualitatively and quantitatively highlighted the dependence of osseodensification instrumentation on presence of trabecular volume, it also demonstrated positive healing outcomes in its use with limited trabeculae, with no indications of microdamage at either healing time (3 or 12 weeks). This warrants future studies that directly compare osseodensification instrumentation to conventional subtractive techniques, specifically in the absence of trabecular bone volume at the osteotomy site prior to implant placement.

In conclusion, the histomorphometric evaluation of osseodensification instrumentation in the presence of trabecular bone volume (Exp.) exhibited improved healing outcomes relative to sites with limited trabeculae (Neg. Ctrl). Nonetheless, osseodensification drilling had no negative influence such as necrosis, inflammation, or dehiscence on bone healing, with no indications of microdamage or microfractures. In a systematic review from 2024, Kalra et al. reported that human clinical research pertaining to osseodensification usage has produced predictable and positive effects [36]. However, despite a number of research investigations, the existing literature mostly consists of studies on animals or clinical cases with short-term follow-ups [21]. One contributing factor to this could be attributed to the novelty of the drills used for osseodensification, which are still not widespread in standard clinical practice [37]. However, this demand is expected to rise in tandem with upcoming in vivo research which could focus on evaluating the effect of osseodensification instrumentation on bone healing through long-term follow-ups [37].

## Data Availability

The original contributions presented in the study are included in the article, and further inquiries can be directed to the corresponding author.
